# Snapping of the Subacromial Bursa: A New Cause of Shoulder Pain Demonstrated with Dynamic Ultrasound

**DOI:** 10.3390/biomedicines13040766

**Published:** 2025-03-21

**Authors:** Arnaud Delafontaine, Raphaël Guillin, Mickael Ropars, Philippe Collin

**Affiliations:** 1 Human Movement, Adaptation and Sports Performance Team, CIAMS, University Paris-Sud, Université Paris-Saclay, 91405 Orsay, France; 2 Laboratoire d’Anatomie Fonctionnelle, Faculté des Sciences de la Motricité, Université Libre de Bruxelles, 1050 Bruxelles, Belgium; 3 Laboratoire d’Anatomie, de Biomécanique et d’Organogenèse, Faculté de Médecine, Université Libre de Bruxelles, 1050 Bruxelles, Belgium; 4 Service de Radiologie de l’Hôpital Sud, CHU de Rennes, 16 Boulevard de Bulgarie, 35203 Rennes, CEDEX, France; raphael.guillin@chu-rennes.fr; 5 Département de Chirurgie Orthopédique et Traumatologique, Centre Hospitalo-Universitaire Pontchaillou, 2 Rue Avenue Henri Le Guilloux, 35033 Rennes, CEDEX, France; mickael.ropars@chu-rennes.fr; 6 CHP Saint-Grégoire, 6 Boulevard de la Boutière, 35760 Saint-Grégoire, France; docphcollin@gmail.com; 7 Clinique Victor Hugo, 5 Bis Rue du Dôme, 75016 Paris, France; 8 Hôpital Américain de Paris, 55 Boulevard du Château, 92200 Neuilly-sur-Seine, France

**Keywords:** snapping scapula, shoulder anatomy, subacromial bursa, dynamic sonography

## Abstract

**Introduction.** Compared to pain, weakness, and stiffness, snapping phenomena are less frequently reported. The anatomical implication of subacromial bursa on snapping syndrome has not yet been studied despite of the fact that subacromial volume is implicated in this syndrome. The aim of this study is to analyze the anatomical and dynamic implication of the subacromial bursa in snapping syndrome. **Methods.** We conducted a retrospective of symptomatic case series (n = 9) study including dynamic sonography, video recordings resulting from standardized clinical dynamic examinations, and the results of shoulder magnetic resonance imaging. Nine patients complaining of snapping phenomena of the anterior shoulder (seven males and two females, mean age: 37.1 ± 10.2 years old), in whom dynamic sonography could confirm the diagnosis of snapping subacromial bursa, were included in this study. **Results.** All the patients included in this study presented non-traumatic painful snapping syndrome without plication before the snap on the dynamic sonography. All complained of a disabling snap of the shoulder associated with pain and without folding before the snapping phenomenon. Four of them had a bursitis of the subacromial bursa diagnosed on their shoulder’s magnetic resonance imagery. No significant statistical correlation (rS = −0.372; *p* = 0.595) was found between the triggering mechanisms, such as the snap shoulder release position, and the position of the anterior recess of the subacromial bursa relative to the biceps’ tendon. **Conclusions.** This study highlights the anterior recess of the subacromial bursa as a previously underexplored anatomical contributor to snapping syndrome, particularly in young, physically active individuals, emphasizing the need for dynamic sonography in diagnosing this condition. The anterior recess of the subacromial bursa represents an additional cause of snapping, which especially takes place in young and physically active patients. More than sport practice, professional activities that require repetitive tasks of the shoulder seem to represent a risk factor.

## 1. Introduction

Compared to pain, weakness, and stiffness, snapping phenomena are less frequently reported [1,2,3]. Such symptoms, resulting in repeated popping sensations during motion of the joint, may result in significant discomfort with the limitation of sports-related or professional activities [4]. In the scapulothoracic joint, abnormal behavior of the scapula during motion of the shoulder girdle may result in abrupt movement of the bone [2], a condition named “snapping scapula” [3,5,6].

Scapulothoracic joint dysfunction can be due to unusual osteochondroma [7,8] or intramuscular myxoma [9]. “Snapping scapula” was firstly described in 1867 [10] and corresponds to an audible or palpable pop of the scapula which occurs during the scapulothoracic joint’s movement.

More laterally, the glenohumeral joint also hosts various intra-articular or extra-articular causes of snaps [1,3], articular cartilage, loose bodies, bicep tendon, isolated brachialis muscle rupture [11], subluxation of an accessory coracobrachialis [12], rotator cuff [13] or glenoid labrum tears [14], anatomical variants or impingement of the subacromial bursa [15], scapulothoracic [16], or subacromial subdeltoid bursitis [17]. The latter condition has only been reported in one patient in whom a clue to diagnosis was the success of bursectomy without true dynamic demonstration [18]. However, the anatomical implication of subacromial bursa on snapping syndrome has not yet been studied. The concept of “snapping of the subacromial bursa” refers to the rapid sliding or displacement of the anterior recess of the subacromial bursa beneath the coracoacromial ligament during shoulder movement, producing an audible or palpable snap. This phenomenon is distinct from other shoulder snapping syndromes, such as those associated with tendon movement over bony structures, like the rotator cuff or the bicep tendon [19,20]. Only one radiological study [21] using ultrasonography has anatomically demonstrated the presence of a smaller subacromial volume for patients with shoulder impingement syndrome. This reduction in subacromial volume could be due, in part, to the combined anatomical morphology of the lateral acromion and greater tuberosity [22].

In recent studies, dynamic sonography has been increasingly used to investigate subacromial structures in shoulder pathologies, particularly in the context of impingement syndrome [23]. However, despite the growing understanding of the role of the subacromial bursa in these conditions, its specific involvement in snapping syndrome remains underexplored. This study aims to fill this gap by providing a detailed anatomical and dynamic assessment of the subacromial bursa’s contribution to snapping phenomena, particularly in young, physically active patients.

The main hypothesis is that impingement of the anterior recess of the subacromial bursa between the coracoacromial arch and the rotator interval is abrupt in the course of snapping syndrome [24,25]. To test our hypothesis, we employed dynamic sonography combined with standardized clinical examinations and shoulder MRI, which allowed for a comprehensive evaluation of the subacromial bursa’s anatomical behavior and its role in the snapping phenomenon. By analyzing the motion of the anterior recess of the bursa in relation to the coracoacromial ligament, we were able to observe the dynamic interactions that lead to snapping, thus providing crucial evidence to support our hypothesis that impingement of the subacromial bursa is a significant contributor to snapping syndrome.

## 2. Materials and Methods

### 2.1. Design

We conducted a multicenter (site 1: Saint Grégoire Hospital, France; site 2: University Center Hospital Rennes, France) retrospective symptomatic case series (n = 9) study including dynamic sonography, video recordings resulting from standardized clinical dynamic examinations, and results of shoulder magnetic resonance imaging (MRI) made at time of the study. This study was approved by the local ethic committee of “Vivalto Santé” (IRB registration number: CERC-VS-2024-05-1, approved on 17 May 2024). In line with the declaration of Helsinki, all participants provided their written consent after being informed about the nature and purpose of this study.

### 2.2. Inclusion and Exclusion Criteria

The selection criteria for patients included individuals with symptomatic anterior shoulder snapping, confirmed by dynamic sonography, and without a history of shoulder trauma or surgery. The exclusion criteria included patients with significant rotator cuff tears, major structural shoulder abnormalities, or previous shoulder surgeries, as these factors could confound the results of the study.

### 2.3. Sonography and Video Recording Protocol

Previous studies have used dynamic sonography to investigate various shoulder pathologies [26], demonstrating the utility of sonography in assessing subacromial impingement and related shoulder disorders [27]. Our study builds upon this methodology by specifically focusing on the dynamic interactions between the subacromial bursa and the coracoacromial ligament, providing new insights into its role in the snapping phenomenon.

We applied the same methodology used in previous published studies [28,29]. All video recordings and dynamic sonography were performed by one experienced musculoskeletal sonographer (i.e., GR, 15 years of experience in musculoskeletal imaging) and are available for review. Dynamic sonography was performed using a Philips iU22 scanner with a 12 MHz linear-array transducer (Philips Medical Systems, The Netherlands), which had been previously validated for musculoskeletal imaging [30,31]. This equipment has been widely used in the assessment of shoulder pathologies, providing high-resolution images necessary for the accurate visualization of the subacromial bursa during dynamic movements.

All sonography examinations included an initial static study, with the patient’s shoulder in a resting position, and a dynamic study.

**Figure 1:**
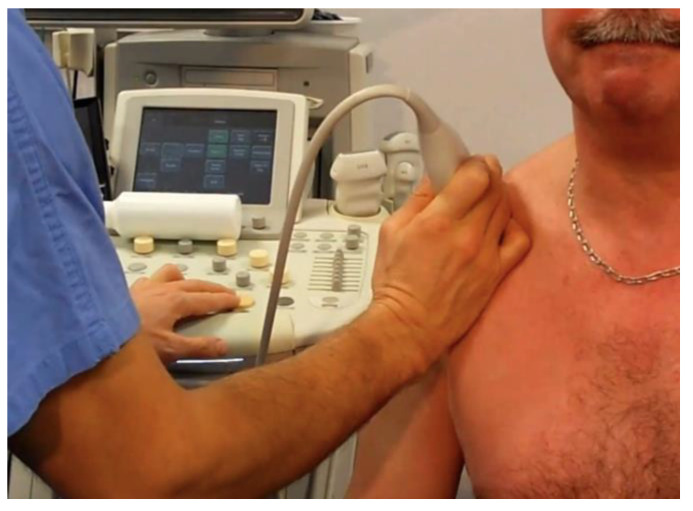
Illustration showing the position of the sonographic probe applied to all patients, successively. This position aims to place the acromial bone posteriorly in the image and explore the relationship between the coracoacromial ligament on one side and the rotator interval and long bicep tendon on the other. Motion is then applied to the shoulder in order to reproduce the snaps.

The subacromial bursa was assessed for signs of bursitis (i.e., synovial thickening or fluid in the bursa). The dynamic part of the sonography study was performed with the transducer in a sagittal or slightly sagittal oblique position, in a manner which placed the acromion and coracoacromial ligament in the posterior half of the view and the rotator interval in the anterior half of the latter, thus allowing us to explore their relationship (Figure 1 and video available in Supplementary Materials).

Patients were then asked to mobilize the shoulder in order to reproduce the snap encountered in their daily practice. The videos were reviewed for the subacromial bursa motion and the relationship of the subacromial bursa with peripheral muscles and adjacent bone landmarks.

### 2.4. Symptomatic Patients

Nine patients complaining of snapping phenomena of the anterior shoulder (seven males and two females, mean age: 37.1 ± 10.2 years old) were referred for dynamic sonography. Patients had been previously examined by one of the two orthopedic surgeons involved in this study in order to clinically exclude other usual causes of snapping shoulder, including snapping scapula syndrome or glenohumeral, sternoclavicular, or acromioclavicular instability. During sonographic examination of the shoulder, the patients were asked to repeatedly reproduce their snaps while the probe was placed in the sagittal plane in order to analyze the behavior of the subacromial bursa, on one side, relatively to anterior edges of the acromion and coracoacromial ligament on the other side. In the meantime, the involvement of other tendinous, ligamentous, and bony structures of the anterior shoulder was also ruled out. When snaps of the subacromial bursa were demonstrated, special attention was also paid to the deep fibers of the deltoid muscle lying in close vicinity.

**Figure 2:**
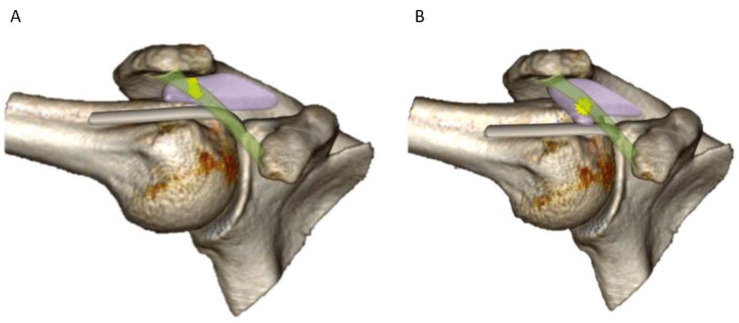
Illustration of the snapping of the subacromial bursa. (**A**) During external rotation of an abducted shoulder, the anterior recess of the subacromial bursa lays posteriorly to the coracoacromial ligament. (**B**) When applying an internal rotation, the anterior recess abruptly glides anteriorly underneath the coracoacromial ligament, thus provoking a snap. Such a snap may also occur when an external rotation is consecutively applied to the shoulder due to the sudden return of the anterior recess posteriorly to the ligament.

All the patients in whom dynamic sonography could confirm the diagnosis of snapping subacromial bursa were included in this study. Such a diagnosis was considered when the anterior recess of the bursa, traveling back and forward underneath the coracoacromial ligament, was brought to abruptly impinge with the anterior edge of the latter, thus producing a snap (Figure 2A,B and Supplementary Materials).

An additional case, wherein an impingement with the coracoacromial ligament did not involve the subacromial bursa but a unstable flap tear of the supraspinatus tendon, was not included in this study. Similarly, eighteen patients in whom dynamic sonography ruled out the involvement of the anterior subacromial bursa were not included in this study.

Videos demonstrating the involvement of the subacromial bursa in the snaps were recorded and retrospectively analyzed, while the ranges of motion generating the snaps were also noted (i.e., for the videos, please see Supplementary Materials).

Among the nine patients with snapping subacromial bursa, six were operated. The result was considered excellent in five or six in whom the symptoms disappeared, allowing the patients to return to work without pain or discomfort. The remaining operated patient became free of snaps but kept complaining of disability. It is of notice that this patient had, at the time of surgery, an especially consistent bursitis of the shoulder. Three patients with acceptable disability were not operated and were proposed conservative treatment.

### 2.5. Statistical Analysis

A Spearman rank correlation analysis was performed to assess the relationship between the triggering mechanisms, the snap shoulder release position, and the position of the anterior recess of the subacromial bursa relatively to the bicep tendon. The threshold of significance was set to *p* < 0.05.

## 3. Results

**Figure 3:**
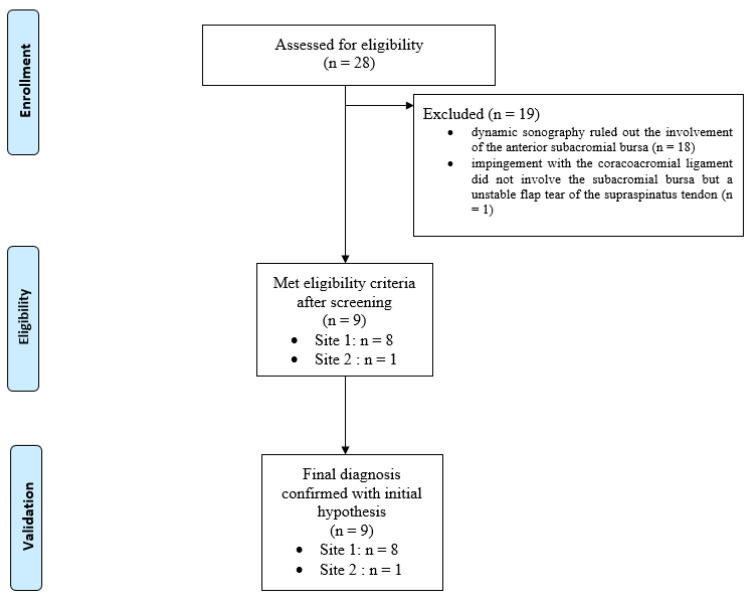
Flow chart diagram of the case series study.

**Figure 4:**
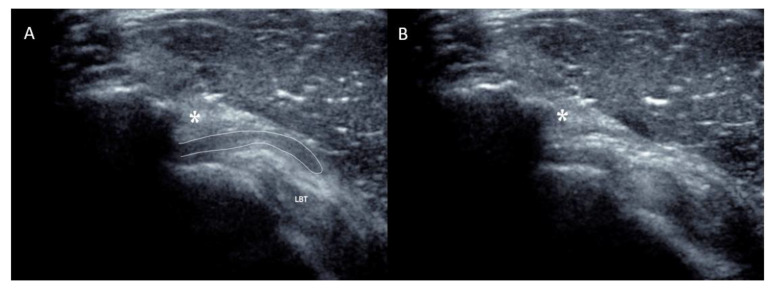
Sagittal view of the shoulder along the anterior edge of the acromion as demonstrated in Figure 1. (**A**) During slight antepulsion of the shoulder, the anterior recess of the subacromial bursa is visible between the coracoacromial ligament (asterisk) and the deltoid muscle superficially and the rotator interval and long bicep tendon (LBT) in a deeper position. (**B**) During retropulsion of the shoulder, anterior recess of the subacromial moves backward and suddenly disappears underneath the coracoacromial ligament and neighboring acromion, thus provoking an audible snap.

The nine patients included in this study (Figure 3) presented non-traumatic painful snapping syndrome without plication before the snap upon dynamic sonography (Figure 4A,B and video available in Supplementary Materials).

Four patients had a bursitis of the subacromial bursa diagnosed upon shoulder MRI (Table 1). The main characteristics of the patients are summarized in Table 1.

All patients complained of a disabling snap of the shoulder associated with pain and without folding before the snapping phenomenon.

The results concerning the relationship between the triggering mechanisms, such as the snap shoulder release position, and the position of the anterior recess of the subacromial bursa relatively to the bicep tendon showed a moderate negative correlation (rS = −0.372; *p* = 0.595), indicating a slight trend for the distance to decrease as the triggering mechanisms changed. However, this correlation was not strong, suggesting that other factors may have contributed to the variability observed in the distance measurements. The analysis showed no statistically significant correlation (*p* = 0.595).

## 4. Discussion

To better situate our findings within the context of the existing literature, we have compared our results with the few studies that have explored snapping phenomena in the shoulder. Previous research has documented various causes of shoulder snapping, with a primary focus on the glenohumeral, scapulohumeral, and sternoclavicular joints [1,2,3]. However, the role of the subacromial bursa, particularly its anterior recess, as an additional cause of snapping syndrome has been sparsely reported. Our study demonstrates that the anterior recess of the subacromial bursa, which has been largely neglected in previous anatomical studies [31], plays a crucial role in the snapping phenomenon. This finding is consistent with Birnbaum et al. [32], who noted the potential of the subacromial bursa’s superficial sheet to fold during shoulder abduction, but differs in that we observed no folding and instead found the bursa sliding under the coracoacromial ligament. These variations in observations emphasize the need for further investigation into the exact role of the subacromial bursa in snapping syndrome. Furthermore, our results support findings from studies such as that of Stallenberg et al. [33], who reported thickening of the anterior subacromial bursa in shoulder pain syndromes. However, we extend these findings by showing that this anatomical variation is not only associated with subacromial impingement but may also directly contribute to snapping phenomena.

In all cases of our study, snaps of the subacromial bursa were demonstrated by dynamic sonography to result from the sudden sliding of both deep and superficial sheets of the subacromial bursa underneath the subacromial arch. As a matter of fact, the whole anterior recess of the bursa, appearing to be firmly attached to the surface of the rotator interval, was shown to disappear under the coracoacromial ligament during motion of the shoulder, thus leading to the snap. During the opposite movement of the shoulder, the anterior recess was brought forward to reappear, leading to another snap of various intensities. The maneuver spontaneously shown by the patients in order to generate the snaps consisted in 90° antepulsion (n = 1), 45° retropulsion (n = 1), or, in the remaining seven cases, a 90° abduction of the shoulder added to the internal and external rotation of the joint. In only one case, wherein the snaps resulted from the pure abduction of the shoulder, lateral but not anterior recess of the subacromial bursa was shown to abruptly glide under the acromion, while the coracoacromial ligament was not involved.

In four cases, slow-motion analysis of the videos demonstrated moderate deep fibers of the deltoid muscle neighboring the involved bursal sheet to move synchronously during the snap (Table 1). In such cases, this led us to consider the anatomical attachment between the two structures to be firm, while it was much looser in other cases. The position of the recess relative to the middle of the biceps’ tendon is summarized in Table 1. In this study, dynamic sonography was chosen as the primary imaging technique, as it is the only method capable of evaluating shoulder impingement and catching phenomena in real time, contrary to MRI. MRI was used primarily to assess bursitis, and no additional lesions were detected. Future studies could compare dynamic sonography with other imaging modalities to explore their complementary roles. Additionally, dynamic examination cannot be replicated during arthroscopy, for example, due to the patient’s anesthetized state. Six patients in our cohort who underwent exploratory arthroscopy with bursitis treatment showed no lesions other than bursitis, consistent with the ultrasound findings. The six patients who underwent surgery progressed without complications and demonstrated satisfactory clinical outcomes one month postoperatively.

Snaps occurring within the shoulder may result from a wide spectrum of structures [1,2,3]. Among them, glenohumeral, scapulohumeral, and sternoclavicular joints represent classical causes, which are usually easy to diagnose on the basis of clinical examination. Concurrently, multiple snaps occurring on the anterior and lateral edge of the shoulder are usually more difficult to understand through clinical inspection and palpation due to the fact that they lie deeper in the bulge of the deltoid muscle.

Causes of snaps of the anterior shoulder have been scarcely reported in the literature [1,3]. They include impingements between an accessory slip of the pectoralis major and the coracoid process, between the subcoracoidal bursa and the latter’s bony structure, and between an unstable flap of the supraspinatus tendon and the coracoacromial ligament [34]; the latter was only encountered in one case in the present study.

Our study demonstrates the anterior recess of the subacromial bursa to represent an additional cause of snapping, which especially takes place in young and physically active patients. More than sport practice, professional activities that require repetitive tasks of the shoulder seem to represent a risk factor. In all of our patients, shoulder discomfort related to snaps had led to temporary breaks from work and had required clinical assessment by an orthopedic surgeon.

As documented in other joints, such as the hip joint [29], dynamic sonography allowed us to gain relevant understanding of the pathophysiology of anterior snapping syndrome. The mechanism that generated the snaps required the patients to oppose the anterior recess of the subacromial bursa to the overlying coracoacromial ligament, a situation obtained in most cases through an 90° abduction with internal/external rotation of the shoulder [18]. In a previous study, Birnbaum et al. [32] demonstrated the ability of the superficial sheet of the subacromial bursa to fold in an accordion-like pattern up to 50° abduction while it slides and to invaginate under the coracoacromial arch above 50° abduction [27,35]. According to the authors, however, the lateral edge of the superficial sheet at the level of the peripheral recess of the bursa cannot invaginate itself due to the fact it is, in numerous cases, firmly adherent to both the coracoacromial ligament and the deep fascia of the deltoid muscle. Numerous findings demonstrated by sonography in our patients with snapping subacromial bursa differed from previous observations [27,35]. Firstly, no folding of the superficial sheet of the bursa was encountered in the whole process of abduction of the shoulder. In our opinion, the fact that, due to the need for the anatomic exposition of the bursa, deltoid fibers were detached from the superficial sheet may have allowed an artificial folding of the latter that is not encountered in the living [36]. Secondly, in all our patients, both the superficial sheet of the subacromial bursa and its anterior recess disappeared under the coracoacromial ligament during 90% abduction with external rotation, suggesting the former two to be free of any attachment in the field of subacromial bursa snapping syndrome. We believe that the lack of attachment of the anterior recess of the subacromial bursa to the deltoid muscle at the level of the rotator interval represents the main risk factor for the syndrome to occur.

The fact that, at 90° abduction, the anterior recess of the subacromial bursa is located in close vicinity to the anterior aspect of the acromion and the neighboring coracoacromial ligament also contributes to the snapping phenomenon. In all cases in the present study, the fact that the anterior recess of the subacromial bursa was located superficially to the rotator interval contributed to the snapping phenomenon, as the long biceps’ tendon appeared to lie directly underneath the acromion and the neighboring coracoacromial ligament at 90° abduction. During the subsequent internal and external rotation of the shoulder, both the tendon and the bursa were shown to glide under the coracoacromial arch, thus producing the snaps. However, its location may vary and lie more anteriorly according to Mitchell et al. [37] and Stallenberg et al. [33]. Birnbaum and Lierse [23] stated that the anterior recess of the subacromial–subdeltoid bursa rests along the coracohumeral ligament. As this location was encountered in all our patients, one may consider it as a significant contributing factor to snapping syndrome.

Another explanation for snapping of the anterior recess of the bursa may lie in the short distance between the surface of the rotator interval and the subsurface of the coracoacromial ligament.

Because dynamic sonography had demonstrated the involvement of the anterior subacromial bursa in the annoying snaps of the shoulder, surgeons were able to perform an arthroscopic subacromial decompression with a high confidence in the clinical results, a situation which is less likely when the cause for the snap has not been determined preoperatively. Despite the fact that one patient with major bursitis kept complaining of pain of the shoulder in the postoperative period, all other operated patients reported the complete disappearance of both pain and snapping phenomena.

In our study, four patients were diagnosed with bursitis, though its role in the snapping phenomenon remains uncertain. The existing literature suggests that bursitis, particularly subacromial bursitis, may contribute to the development of snapping symptoms, either by increasing friction between the bursal layers and the coracoacromial ligament or by causing an alteration in the normal anatomical movement of the bursa during shoulder motion. However, it is important to note that bursitis can also occur independently of snapping syndrome. Studies such as those by Chang et al. [19] and Daghir et al. [20] indicate that subacromial bursa thickening or inflammation, as seen in the impingement syndrome, does not always result in snapping. These findings suggest that, while bursitis might exacerbate or facilitate the snapping phenomenon, its presence does not necessarily imply a causal relationship. Future studies with larger sample sizes and more detailed clinical assessments are needed to clarify the exact role of bursitis in snapping syndrome.

Impingement of the anterior recess of the subacromial bursa between the coracoacromial arch and the rotator interval was abrupt in the course of snapping syndrome demonstrated in the present study. However, we believe that smoother impingement, without a snap, may occur and favor the suffering of the anterior bursa in the course of the so-called « subacromial impingement syndrome » described by Neer in the 1970s [35]. From a static point of view, Stallenberg et al. [33] demonstrated the association between the anteromedial pain of the shoulder and thickening of the anterior subacromial bursa with sonography. If the author stated this finding to be associated with subcoracoidal impingement, we wonder if at least partial association to subacromial impingement may not be considered.

The use of dynamic sonography, as “subacromial impingement imaging test”, in order to better understand the subacromial impingement phenomenon (i.e., intrinsic and extrinsic factors) has been proposed by Bureau et al. [27]. It could usually be integrated, without difficulty, as a routine sonography shoulder examination protocol.

Contrary to Beals et al. [25], in our study (see Table 1), the recess’ shift in the subacromial bursa was not associated with a plication before the snap during dynamical sonography. We noticed that the effusion of the subacromial bursa led to intussusception later and was, therefore, not an a priori risk factor for snapping syndrome. In fact, Bouju et al. [36] did not find any correlation between ultrasonography results and the effectiveness of a local anesthetic injection into the subacromial bursa. These results propose that ultrasound irregularities might simply reflect anatomical variations, aligning with prior research in asymptomatic individuals. Consequently, there could be an excessive diagnosis of subacromial impingement syndrome. This study presents some limitations: (i) the analysis of the deep insertion of the deltoid, as this could have been loose in some patients and might have contributed as a factor favoring snapping syndrome; (ii) the evaluation of the shoulder surgery’s effectiveness, notably subacromial bursectomy, in the therapeutic management of snapping syndrome; and (iii) the small sample size (n = 9), which may have restricted the generalizability of our findings to a larger population. While the small sample size limits statistical power, the data obtained provide valuable insights into the anatomical and dynamic role of the subacromial bursa in the snapping phenomenon, warranting further investigation in larger cohorts. Another limitation of this study was (iv) the absence of a control group, which made it difficult to determine whether the observed findings were specific to snapping syndrome or simply represented anatomical variations. Future studies with a control group are necessary to better differentiate between condition-specific findings and normal anatomical variations.

While our study suggests that the anterior recess of the subacromial bursa plays a key role in snapping syndrome, it is essential to consider other potential contributing factors. Previous studies indicate that thickening of the subacromial bursa and dynamic changes in its structure can also be associated with shoulder impingement and snapping phenomena. Chang et al. [19] demonstrated that subacromial bursa thickening, detected via sonography, is a relevant feature in shoulder impingement syndrome. Furthermore, Daghir et al. [20] highlighted the importance of dynamic ultrasound in understanding the movement of the subacromial–subdeltoid bursa, which could also impact the development of snapping symptoms. Therefore, other anatomical factors, such as interactions with the deltoid muscle or the coracoacromial ligament, should also be considered in future studies.

In this study, while professional activities involving repetitive shoulder movements were suggested as a potential risk factor for symptomatic snapping, the absence of statistical analysis limited our ability to draw definitive conclusions regarding this association. Future studies with larger sample sizes and appropriate statistical methods, such as regression analysis, would be required to better understand the impact of these activities on the development of snapping syndrome.

## 5. Conclusions

The subacromial bursa appears in the literature as a neglected tissue [38]; nevertheless, the subacromial–subdeltoid bursa seems to be implicated in shoulder pain [39,40]. Repetitive asymptomatic [26] and symptomatic shoulder snaps must be considered important clinical symptoms and taken into account with higher consideration during patients’ clinical examination by surgeons, physiotherapists, and other clinicians [41]. The etiology of symptomatic shoulder snapping is often difficult to find and must be systematically searched for using dynamic sonography [1]. Our study demonstrates the implication of the anterior recess of the subacromial bursa as an additional etiology of snapping, more particularly in young and physically active patients. Treatment protocols should include tailored rehabilitation strategies, focusing on reducing inflammation, restoring shoulder stability, and addressing any associated rotator cuff pathology.

Future research, including clinical controlled randomized studies, is necessary to better understand the anatomical and clinical role of the subacromial bursa in snapping syndrome, which could be improve the surgical management of nearby reparable rotator cuff injuries [42] and physical therapy [43,44].

In addition to the clinical implications, future research should focus on larger, multicenter studies to confirm these results and examine the effectiveness of different therapeutic approaches for subacromial bursa-related snapping syndrome. Furthermore, the use of dynamic sonography to monitor treatment progress may offer significant benefits. As highlighted by Zhao et al. [45], future systematic reviews and meta-analyses will further inform clinical practice and guide the refinement of management strategies for this condition.

## Figures and Tables

**Table 1:** Main characteristics of symptomatic patients.

Subjects	Sex	Age	Occupation Reported to Generate the Snaps	Snap Shoulder Release Position	Sonographic Position of the Anterior Recess of the Subacromial Bursa Relatively to the Bicep Tendon	Shift in the Recess of the Subacromial Bursa upon Sonography	Attraction of Deltoid’s Fibers on Sonography
1	male	52	caregiver	retropulsion/neutral	15 mm back	yes	none
2	male	24	meat boner	abduction 90° IR/ER	posterior edge	yes	moderate
3	male	36	truck driver	abduction 90° IR/ER	posterior edge	yes	moderate
4	female	40	laboratory technician	anterior elevation	5 mm forward	yes	light
5	male	40	chef cook	abduction 90° IR/ER	middle part	yes	none
6	female	21	horse riding	abduction 90° IR/ER	5 mm forward	yes	none
7	male	48	police officer	abduction 90 pure	5 mm forward	yes	light
8	male	41	storekeeper	abduction 90° IR/ER	10 mm back	yes	none
9	male	32	house painter	abduction 90° IR/ER	10 mm back	yes	none

ER: external rotation; IR: internal rotation; and mm: millimeters.

## Data Availability

The datasets used and/or analyzed during the current study are available from the corresponding author upon reasonable request.

## Supplementary Materials

The following supporting information can be downloaded at: https://www.mdpi.com/article/10.3390/biomedicines13040766/s1.
